# Light Emitting Diode (LED) Phototherapy versus Conventional Phototherapy in Neonatal Hyperbilirubinemia: A Single Blinded Randomized Control Trial from Coastal India

**DOI:** 10.1155/2019/6274719

**Published:** 2019-04-11

**Authors:** Sreesravya Gutta, Janardhan Shenoy, Sowmini P. Kamath, Prasanna Mithra, B. Shantharam Baliga, Muralikeshava Sarpangala, Mukund Srinivasan

**Affiliations:** ^1^Hope Children Hospital, Hyderabad 500029, Telangana, India; ^2^Department of Pediatrics, Kasturba Medical College, Mangalore, Manipal Academy of Higher Education, Manipal, Karnataka, India; ^3^Department of Community Medicine, Kasturba Medical College, Mangalore, Manipal Academy of Higher Education, Manipal, Karnataka, India; ^4^Department of Pathology, University of Texas Medical Branch, Galveston, Texas, USA

## Abstract

Neonatal hyperbilirubinemia is a common problem with potentiality to cause irreversible brain damage. Reduction of serum bilirubin level is essential to minimize such damage. Compact fluorescent tubes, halogen bulbs, fiber optic blankets, and LEDs are commonly used light sources for phototherapy with varying efficacies. This study aimed at evaluating the effect of LED versus conventional phototherapy on (a) rate of reduction in total serum bilirubin levels, (b) effect on urinary lumirubin excretion, and (c) comparing side effects of phototherapies among neonates with hyperbilirubinemia. In this randomized control trial, 166 neonates ≥ 35 weeks of age requiring phototherapy were recruited and further divided into 2 groups [LED (83) and conventional (83)] by using computer generated random numbers. Serial total serum bilirubin levels and random urinary lumirubin levels were collected and side effects of phototherapy were noted. Rate of fall in total serum bilirubin levels (TSB, *μ*mol/L/hour) and random urinary lumirubin levels were computed. Data were collected using a pretested proforma. Analysis was done with Statistical Package for Social Sciences (SPSS) version 11.5. Independent sample “t” test and Chi-square tests were used with p value of <0.05 being significant. Significant difference was documented in mean rate of decrease of TSB (*μ*mol/L/hour) in LED group (5.3 ± 2.91) when compared to conventional group (3.76 ± 2.39) (p <0.001). A significant increase in mean random urinary lumirubin levels (arbitrary units) was observed in LED group (129.01 ± 33.18) when compared to conventional group (114.44 ± 44.84) (p = 0.021). Side effects were minimal and comparable in both groups. This study concludes the rates of decrease in total serum bilirubin levels and increase in urinary lumirubin levels were significant with LED when compared with conventional phototherapy, implying LED to be more efficacious.

## 1. Introduction 

Neonatal jaundice, a condition that requires etiological evaluation and prompt treatment, is one of the most common issues of neonates. Incidence of neonatal jaundice in India varies from 54.6% to 77% [1]. In most of the neonates, it is just a benign transient phenomenon known as physiological jaundice. Although mostly physiological, sometimes it can result in irreversible bilirubin encephalopathy if not recognized [2], which becomes the concern factor for a developing brain. Hence treatment should be expedited. Out of various treatment modalities available for neonatal jaundice, phototherapy is widely used. During phototherapy, the neonate skin is exposed to a light source of specific wavelength which aids in decreasing bilirubin levels. Thus it has reduced the number of exchange transfusions and bilirubin induced neurologic dysfunction (BIND) [3].

Conventional phototherapy uses compact fluorescent (CFL) or halogen lamps. Light Emitting Diode (LED) is being used as light sources for phototherapy with unique characteristics of portability, power efficiency, lesser heat production, and durability [4].

Few of such comparative studies on efficacy of conventional and LED phototherapy done previously were inconclusive [5–9]. However the Cochrane review in 2011 concluded that phototherapy with either LED light source or conventional light sources decreased serum total bilirubin levels at similar rates [2].

During phototherapy, along with configurational isomer, irreversible structural isomer, lumirubin (2-6%) is derived from bilirubin. During phototherapy, measurement of urinary lumirubin levels accounts for fraction of the total pigment elimination; however, it is a significant contributor as in previous studies [10].

Hence this study was carried out to compare rate of decrease in total serum bilirubin levels and to evaluate the effect of urinary excretion of lumirubin in neonates with jaundice treated either with LED or conventional phototherapy.

## 2. Materials and Methods

### 2.1. Research Design and Study Setting

This randomized control trial was conducted from November 2013 to September 2015, at neonatal care units in tertiary care hospitals attached to Kasturba Medical College, Mangalore, Manipal Academy of Higher Education, Manipal, Karnataka, India. Neonates ≥ 35 weeks and those requiring phototherapy according to Bhutani charts [11] were included in the study. Ventilated babies, preterm neonates (<35 weeks), babies with hypothermia, infants requiring exchange transfusion according to Bhutani charts [11], and culture proven and clinical sepsis were excluded from the study. The trial was registered in clinical trial registry India (CTRI) with trial registration number CTRI/2017/11/010619.

### 2.2. Sample Size

It was calculated assuming combined standard deviation of duration of phototherapy to be 21.4, difference between means of duration of phototherapy to be 11 [6], at 95% confidence interval, 90% power, and 10% nonresponse rate as 166 (83 in each group).

### 2.3. Data Collection Methodology

After obtaining the approval from the Institutional Ethics Committee (IEC), necessary permissions were taken from the hospital authorities. Sequential sampling (nonrandom) technique was used to select the study participants based on the chronological sequence of hospitalization. For the selected study participants, their parents/guardians were approached and explained about the objectives of the study in a language they understood and a participant information letter was provided to them. A written informed consent was obtained from each one of the parent/guardian.

**Figure 1:**
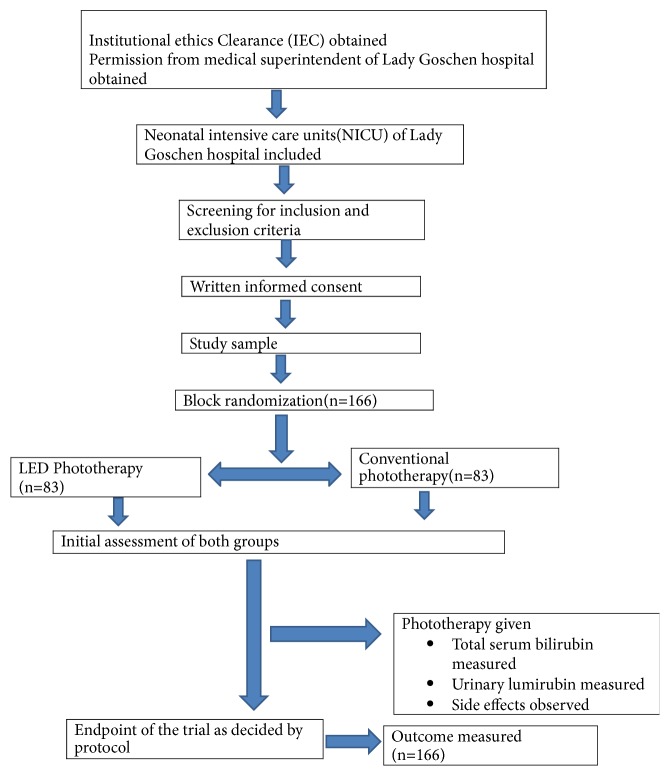
Study flow diagram with the enrolment, allocation, and outcome (CONSORT).

The eligible participants were randomized into two groups using block randomization technique based on computer generated blocks (https://www.sealedenvelope.com/simple-randomiser/v1/lists), into a total of 34 blocks of sizes 4 and 6. Sequentially arranged opaque sealed envelopes were used for the allocation concealment. Study flow is depicted in Figure 1. The interventions were given by the investigator who was not a part of the randomization team and thus they were blinded to the parents of the study participants and the investigators. Data collection was done using a semistructured pretested proforma.

Neonates were kept at 30-40 centimeters from light source and were completely exposed except for genitalia and eyes. In conventional group, the phototherapy equipment had used a combination of alternating four blue and 2 white tube lights (20 W each) that provided irradiance of 8-12 *μ*W/cm^2^/nm with a wavelength of 425-475 nm (Neocare Equipments, Mumbai, India). For LED group, we had used the model Fanem Bilitron sky 5006, which provided irradiance of 30-40 *μ*W/cm^2^/nm with a wavelength of 450 to 500 nm (Fanem Medical Devices, India Pvt Ltd). As per the guidelines of American Academy Pediatrics [11], the LED group met the criteria for intensive phototherapy. This was compared with the available conventional phototherapy units at our neonatal center.

Neonates were given phototherapy, calculated in hours, interrupted only for feeding, cleaning and blood sampling (time taken for these activities were reduced from the calculated duration of phototherapy). Daily weight was recorded and temperature monitoring was done every 6th hourly. Hypothermia was defined if the temperature recordings fell below 36 degree Celsius [12]. Hydration status was assessed based on physical examination and weight monitoring. Dehydration status was defined if there was documented weight loss greater than 5% in a day [13]. Any side effects including skin darkening, rashes, and diarrhea were noted. Tanning of skin under phototherapy was considered as evidence of skin darkening [14].

Under aseptic precautions, a nonfasting venous blood was drawn from neonates into procoagulant vacutainers and serum bilirubin was analyzed by Roche automated clinical chemical analyzer by colorimetric assay. Urine sample was collected within one hour of initiation of phototherapy [10], in plastic neonatal urine collection bags (Minicom), which were shielded from light during collection by diapering the infant. The collected urine samples were kept in containers wrapped with aluminum foil and were frozen [10] till they were analyzed by UV excitable fluorescence method [15]. The baseline data and estimated biochemical values were documented and preserved for data analysis.

Urinary lumirubin levels were determined in 80 out of 83 subjects in LED group and 78 out of 83 subjects in conventional group, since collection of urine was not possible in these neonates within the stipulated time after initiation of phototherapy. Thus for analysis of urinary lumirubin levels, 80 subjects were considered in LED group and 78 in conventional group, respectively.

Urinary lumirubin was analyzed by Grass F et al. [15], where illumination gave a constant increase in lumirubin fluorescence at 415 nm and this reached significance within 60 min. Thus we measured random urinary lumirubin levels within one hour of phototherapy initiation in our study.

Total serum bilirubin levels were measured, till it was reduced to below phototherapy range as per standard protocol [14]. Phototherapy was continued till total serum bilirubin level fell below the phototherapy range or went beyond phototherapy range to exchange transfusion range according to Bhutani charts [11].

### 2.4. Statistical Analysis

The collected data were analyzed using Statistical Package for Social Sciences (SPSS Version 11.5, Chicago IL). Results were presented as mean (SD) and proportions. Independent sample t-test was done to compare the mean difference between LED group and conventional group. Chi-square test was done to find out the associations between categorical variables. A p value of < 0.05 was considered to be statistically significant.

### 2.5. Outcome Measures

The primary outcome measure assessed was the rate of decrease of total serum bilirubin in *μ*mol/L/hour in each group. The secondary outcome measures assessed were estimating urinary lumirubin levels after initiation of phototherapy in each group and to study the side effects of both modalities of phototherapy in neonates.

## 3. Results

Neonates (n=166), who were aged more than 35 weeks of gestation, were included in the study, out of whom 83 each received LED phototherapy (LED group) and conventional phototherapy (conventional group). There was near equal distribution of genders (85 males versus 81 females) in the study. Also, 62% of them had gestational age between 38 and 40 weeks. Birth weight of 2.5 to 3.5 kgs was seen in 64.5% of babies. Blood group incompatibility was documented in 33% of our study population. The mean ages of initiation of phototherapy in both the groups were comparable. The baseline characterstics and the serum bilirubin levels at initiation of phototherapy were not significantly different between the two groups [Table 1].

The mean rate of decrease of serum bilirubin in (*μ*mol/L/hour) was higher in LED group (5.3 ± 2.91) when compared to conventional group (3.76 ± 2.39) and was statistically significant (p <0.001) [Table 2].

The mean levels of random urinary lumirubin (arbitrary units) were higher in LED group (129.01 ± 33.18) when compared to conventional group (114.44 ± 44.84) and this difference was found to be statistically significant (p=0.021) [Table 2].

Side effects such as skin darkening (LED group -3 cases, conventional group -2 cases) and skin rash as transient maculopapular rashes (LED group -1 case, conventional group - 2 cases) were noted. Diarrhea was seen in two cases, (one in each group). Neither hypo/hyperthermia was noted in the study. None of the neonates required exchange transfusion.

## 4. Discussion

With advancement in phototherapy, comparative studies on efficacy of LED and conventional phototherapy for treatment of hyperbilirubinemia have yielded varying results. In this study, we compared the effect of LED versus conventional phototherapy on rate of decrease in total serum bilirubin, urinary lumirubin levels, and their side effects.

We observed a significantly higher rate of decrease in total serum bilirubin with LED. This observation is in line with studies by Karagol BS et al. [6] and EK-isariyaphorn R et al. [16], where they used phototherapy units with irradiances similar to our study. Sherbiny HS et al. [17] documented high intensity LED phototherapy to be better in comparison to intensive conventional phototherapy. However, on the contrary, studies by Mohammadizadeh M et al. (blue fluorescent phototherapy versus LED) [7], Takcı S et al. (intensive conventional and intensive LED phototherapy) [8], and ViauColindres J et al. [18] have concluded that there is no significant difference in rate of decrease in serum bilirubin levels between LED and conventional phototherapy group, since they all had compared between different intensive phototherapy units with matched irradiances as per criteria of American Academy of Pediatrics [11]. In studies by Seidman DS et al. [19, 20] there was no significant difference between phototherapy interventions by blue green LED versus Blue LED versus conventional phototherapy. This was because the LED devices were placed at a distance such that it would provide irradiance of 5-8 *μ*W/cm^2^/nm similar to the conventional phototherapy devices. The consensus statement by Cochrane review in 2011 had reviewed six studies in total. Four studies had matched the irradiances between the units; other two studies had kept the distance between the neonate and the light source to be similar. Cochrane review concluded the rates of decrease of serum total bilirubin are equally efficacious both by LED as well as by conventional (compact fluorescent lamp (CFL) or halogen) light sources of phototherapy [2].

Bilirubin is a metabolic product of heme degradation. Prior to elimination, bilirubin is conjugated with glucuronic acid making it water soluble. In neonates, deficiency of enzyme responsible for this conjugation reaction results in hyperbilirubinemia and phototherapy is the preferred mode of treatment. During phototherapy, the native bilirubin molecule undergoes photochemical reaction resulting in reversible configurational isomer form- 4Z, 15E bilirubin [10, 21], and irreversible structural isomer form known as lumirubin (2-6%) [10, 21, 22]. Lumirubin, being more soluble than bilirubin, is excreted into the bile and urine without conjugation. Lumirubin clearance is known to correlate well with creatine clearance (r = 0.96, p < 0.01) [10]. Thus measurement of urinary lumirubin levels definitely determines treatment efficacy of phototherapy. In this present study, we observed that urinary lumirubin levels collected within one hour of initiation of phototherapy were significantly higher in neonates who received LED phototherapy when compared to conventional phototherapy.

In this study the side effects in both the groups were minimal. However studies by Surmeli-Onay O et al. [23] and Sherbiny HS et al. [17] observed a significantly higher incidence of skin rashes with conventional phototherapy group. All the neonates in our study maintained euthermia under phototherapy. This was similar to the studies by Reddy TR et al. [5] and Uraş N et al. [24].

The present study has few limitations. Comparing between intensive LED and intensive conventional phototherapy as per the current guidelines of American Academy of Pediatrics is essential in our setup.

## 5. Conclusions

To conclude, in our center, LED phototherapy had faster rate of decrease in total serum bilirubin levels and higher excretion of urine lumirubin levels when compared to conventional phototherapy in neonatal hyperbilirubinemia. Side effects were minimal and comparable with both therapies.

## Figures and Tables

**Table 1:** Baseline characteristics of the study population (n= 166).

Characteristics	LED group (n=83) No. (%)	Conventional group (n=83) No. (%)	p value
*Gender*			
Female	37 (47.5)	44 (54.3)	0.277
Male	46 (54.1)	39 (45.9)	
*Parity*			
Primipara	58 (53.2)	51 (46.8)	0.253
Multipara	25 (43.9)	32 (56.1)	
*Blood group incompatibility *	30 (54.5)	25 (45.5)	0.410

	Mean ± SD (Minimum – Maximum)	Mean ± SD (Minimum – Maximum)	

Gestational age (weeks)	38.47 ± 1.34 (35-41)	38.62 ± 1.46 (36-40)	0.330
Birth weight (Kg)	2.87 ± 0.54 (1.5-4)	2.78 ± 0.50 (1.6-4.2)	0.102
Age of initiation of phototherapy (hours)	87.86 ± 27.2 (34-160)	80.80 ± 30.1 (23-181)	0.115
Serum total bilirubin (*μ*mol/L) at initiation of phototherapy	299.93 ± 41.21 (174.42-376.2)	285.74 ± 47.03 (1.12-371.07)	0.075

**Table 2:** Comparison of random urinary lumirubin levels and change scores of serum bilirubin levels between LED and conventional phototherapy (n=166).

Parameters	LED group (n=83) Mean ± SD (Minimum-Maximum)	Conventional group (n=83) Mean ± SD (Minimum-Maximum)	“t” value	p value
Serum bilirubin (*μ*mol/L/hour)	5.3 ± 2.91 (0.68-15.39)	3.76 ± 2.39 (0.22-11.97)	67.72	**<0.0001** **∗**

Urinary lumirubin (au)	129.01 ± 33.18 (50.0-227.0)	114.44 ± 44.84 (50.0-221.00)	2.33	**0.021** **∗**

*∗*: p value significant at 0.05 level.

## Data Availability

The data used to support the findings of this study are available from the corresponding author upon request.
